# The comprehensive strategy in the human health risk assessment of total chromium impurities in cough syrups with Marshmallow Root (*Althaea officinalis*) available in Polish pharmacies: regulatory aspects and special emphasis on Cr(VI) mode of action

**DOI:** 10.1038/s41598-024-56057-7

**Published:** 2024-03-04

**Authors:** Kamil Jurowski, Mirosław Krośniak

**Affiliations:** 1 Laboratory of Innovative Toxicological Research and Analyses, Institute of Medical Studies, MedicalCollege, Rzeszo´w University, Al. mjr. W. Kopisto 2a, 35-959 Rzeszow, Poland; 2 Department of Regulatory and Forensic Toxicology, Institute of Medical Expertises, ul. Aleksandrowska 67/93, 91-205 Lodz, Poland; 3 Department of Food Chemistry and Nutrition, Medical College, Jagiellonian University, Medyczna 9, 30-688 Kraków, Poland

**Keywords:** Analytical biochemistry, Plant sciences, Health care, Medical research, Chemistry

## Abstract

Chromium, which can currently only be considered pharmacologically active and not an essential element, is a very intriguing elemental impurity in final pharmaceutical products, especially traditional herbal medicinal products. This kind of traditional herbal medicinal product with Marshmallow root (*Althaea officinalis* L., radix) registered in the EU is widely used among the European population. The aim of this article is to propose a double regulatory strategy in assessing the human health risk of total chromium impurities in cough syrups with Marshmallow Root (*Althaea officinalis*) available in Polish pharmacies. We applied the strategy based on the requirements of the ICH Q3D (R1) guideline for the assessment of Cr impurities in final traditional herbal medicinal products with Marshmallow Root registered in the EU. Furthermore, we applied the strategy based on the concept of margin of exposure (MoE) considering Cr(VI) genotoxicity mode of action (MOA) and based on BMD_10_ for Cr(VI) as a point of departure (PoD). The total Cr content was in the range: 1.12–9.61 µg/L (in comparison with the ICH Q3D R1 guidelines: 1100 µg/g). Total Cr levels in a single dose were relatively high compared to raw results, but were not a threat to patients. Comparison of estimated results with oral PDE value for Cr in final drugs suggested by the ICH Q3D R1 guideline (10,700 µg/day) show that all the products analyzed were below this value (the highest result was 278.40 ng/day). Despite conservative assumptions, the MoE values obtained for Cr in daily dose for each Marshmallow Root cough syrup were above 10,000; therefore, exposure to Cr would not cause a health risk for specific population groups (children and adults). It can be summarized that each of the phytopharmaceuticals analysed with Marshmallow root available in Polish pharmacies does not represent a health hazard to patients. We confirm the safety of Cr impurities by applying a double regulatory strategy without the application of an expansive and demanding HPLC-ICP-MS technique for Cr speciation.

## Introduction

**Figure 1:**
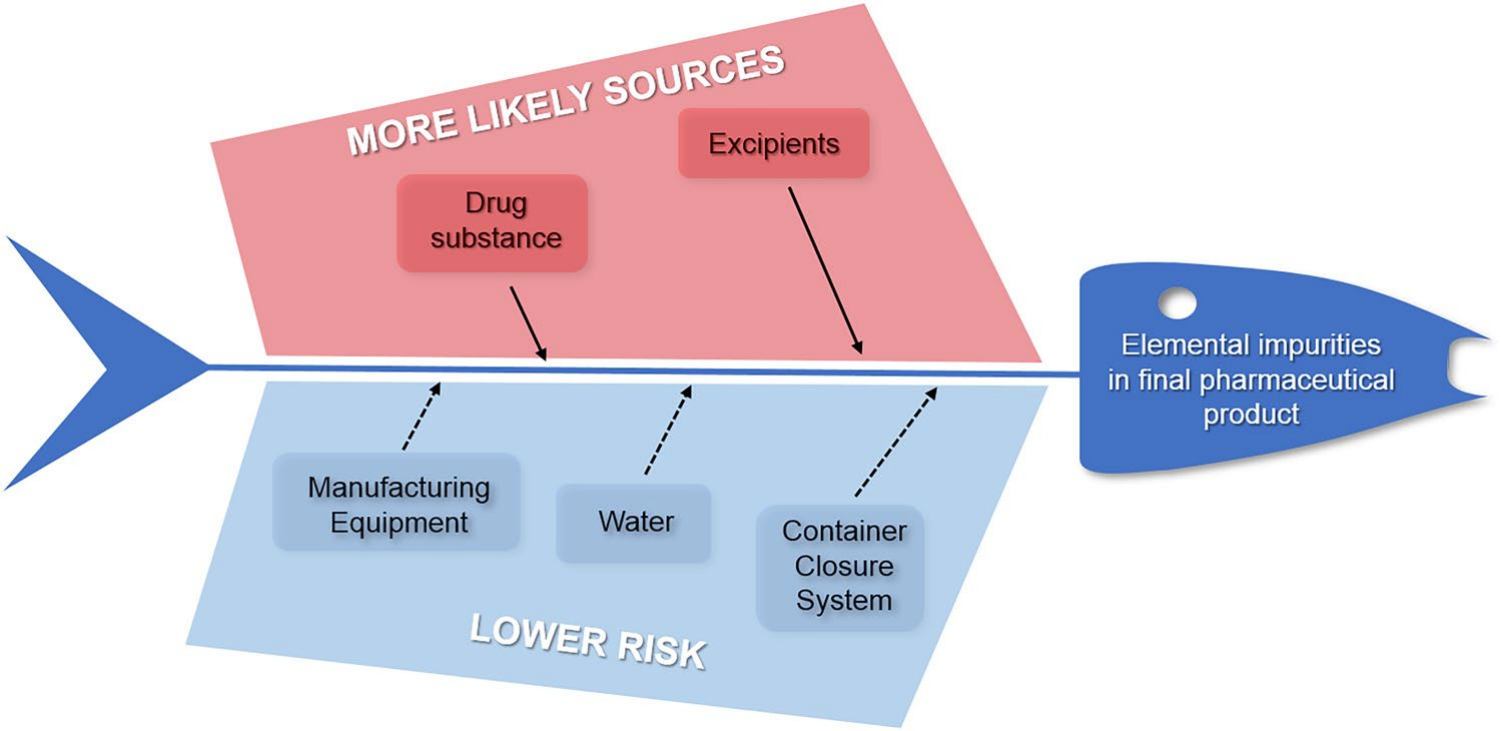
The fishbone diagram for EI sources in final pharmaceutical products.

The oversight and regulation of elemental impurities (EI) within the pharmaceutical sector constitutes a notably crucial and widely discussed subject. Nevertheless, it is often observed that a scarcity of appropriate scholarly literature is evident within this domain. In light of the fact that EI does not confer any discernible therapeutic advantages to patients, meticulous control over their presence in final pharmaceutical formulations is imperative, aligning with established regulatory standards. A pivotal benchmark in this sphere is exemplified by the ICH Q3D (R1) directive pertaining to elemental impurities^1^, which emphatically underscores the necessity for judicious application of human health risk assessment (HHRA) to delineate an ultimate control strategy. When evaluating the production lifecycle of a pharmaceutical product, a limited number of EI sources warrant meticulous attention:

- The active pharmaceutical ingredient (API) embodies residual impurities arising from deliberately introduced elements (e.g., catalyst).
- Excipients or supplementary constituents of the drug product components.
- The manufacturing apparatus (EI that may conceivably infiltrate the drug substance and/or final drug product during the manufacturing process).
- Elemental impurities stemming from solvents (notably water), which are unintentional additions and could potentially be present in the drug substance.
- Container closure systems, encompassing EI that possess the potential to leach into the API and pharmaceutical product from the enclosure system.
- The aforementioned sources of EI, encompassing those of higher likelihood (API, excipients) and lower risk (manufacturing equipment, solvent, container closure system), are succinctly encapsulated in a schematic depiction akin to a fishbone diagram, as represented in Fig. 1.

**Figure 2:**
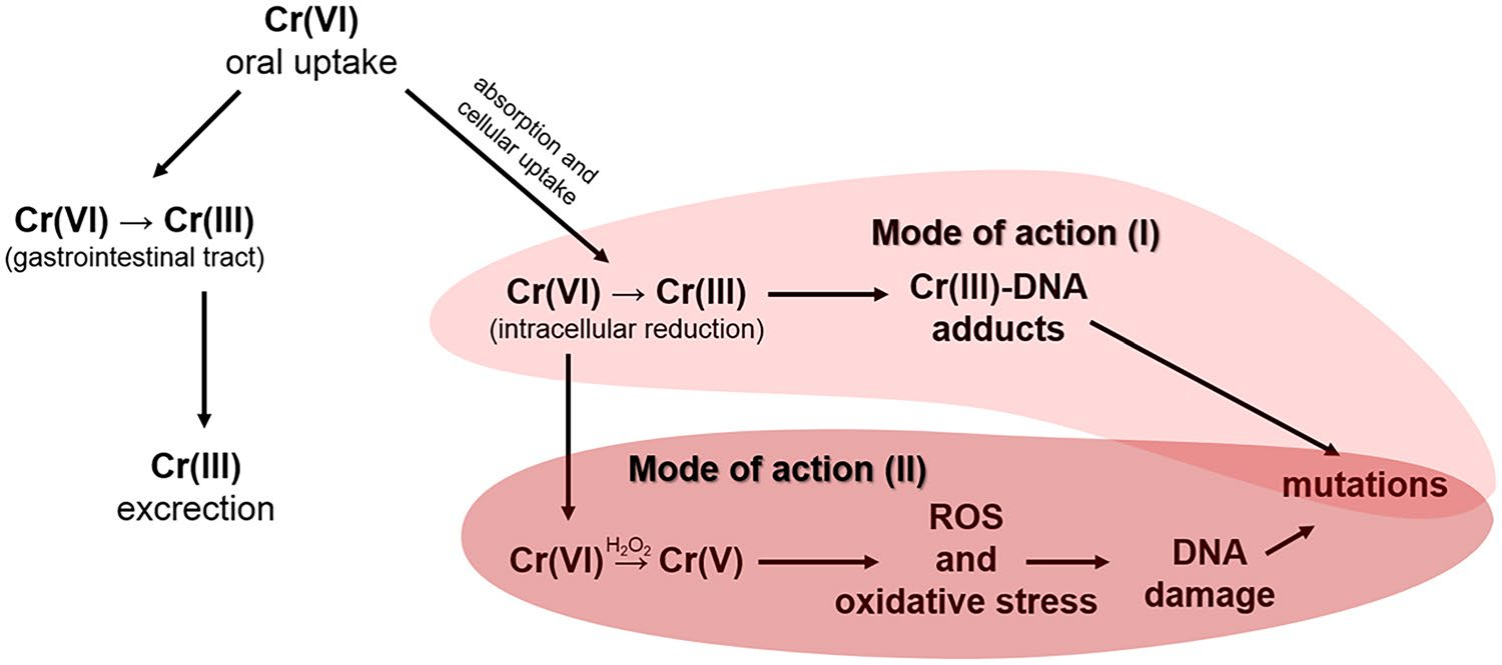
Possible mode of action for Cr(VI) impurities after oral uptake.

An illustrative instance of captivating elemental impurities (EI) within ultimate pharmaceutical formulations is chromium, a constituent currently regarded as solely pharmacologically active and not classified as an indispensable element^2,3^. The pharmaceutical sector exhibits a vested interest in comprehending not only the mechanism of action (MoA) of active ingredient(s) but also their potential adverse effects or reactions^4^. Furthermore, the process of making informed safety determinations, which encompasses the MoA of impurities, necessitates integration into modern toxicological risk assessment protocols^5^. In this specific context, among the diverse array of oxidation states, a handful of forms emerge as particularly significant: Cr(0) (in its elemental form), Cr(II), Cr(III), and Cr(VI)^1^. From a toxicological vantage point, Cr(VI) stands as the most pernicious variant. Delving into the speciation of chromium, it becomes evident that a pivotal driver (primary MoA) behind the genotoxic activity of Cr(VI) (as depicted in Fig. 2) is its intracellular reduction from Cr(VI) to Cr(III). This reduction of Cr(VI) to Cr(III) holds significance even in an earlier phase of the MoA, given its substantial role in determining the bioavailability of Cr(VI) upon oral ingestion. This is particularly crucial due to the potential limitation in the cellular entry of Cr(III) compared to Cr(VI), as the latter faces barriers in traversing cell membranes^6^.

It merits emphasis that following absorption, Cr(VI) undergoes reduction to Cr(III), culminating in the generation of Cr DNA adducts and other forms of DNA damage that lead to mutagenesis^7,8^. This sequence of events is regarded as the principal MoA (Mode of Action I; Fig. 2). The secondary MoA involves the reduction of Cr(VI), yielding Cr(V), which subsequently prompts the generation of reactive oxygen species (ROS) upon reaction with H_2_O_2_. This process engenders the creation of hydroxyl radicals, ROS, and oxidative stress^9,10^, ultimately causing DNA impairment and mutation (Mode of Action II; Fig. 2). It is noteworthy that both modes of action can manifest concurrently and contribute to the genotoxic ramifications attributed to Cr(VI).

This aspect bears significant relevance, particularly within the context of human health risk assessment (HHRA) concerning traditional herbal medicinal products (THMPs). This pertinence is underscored due to the prevalent presence of Cr(VI) in plants, which constitutes an unstable form subject to alteration under standard soil conditions. The availability of Cr(VI) to plants hinges on soil characteristics, primarily soil texture and pH levels. Nonetheless, other forms, including Cr(III) and several intricate Cr anions (such as the well-documented CrO42−), could also be readily assimilated by plants^11^. In the realm of pharmaceutical products, the primary sources of overall chromium impurities encompass residual catalyst elements^12^, pigments employed as colorants^13^, the leaching of substances from equipment or container closure systems, and environmental contamination (a factor of pronounced significance, particularly concerning THMP)^1^.

Of these sources, environmental contamination is relatively unexplored within scientific discourse regarding the safety of THMP, particularly traditional herbal medicinal products. A notable exemplar among Europe’s population is the utilization of extracts derived from *Althaea officinalis* L., radix (Marshmallow root), a THMP that finds widespread application as a demulcent remedy for addressing symptoms associated with oral or pharyngeal cough, including dry cough. Nevertheless, there exists a conspicuous dearth of studies concerning the HHRA of elemental impurities in this category of THMP, readily available in pharmacies. It merits highlighting that all ultimate pharmaceutical products are obligated to adhere to the criteria stipulated within the ICH Q3D (R1) manual pertaining to elemental impurities. Moreover, given that Marshmallow Root cough syrups are targeted towards distinct demographic subsets—children aged 3–6 years, children aged 6–12 years, and adults—an appropriate exposure assessment is imperative.

Furthermore, the formulation of safety determinations encompassing the mode of action of chromium impurities, particularly their neoplastic effects (notably Cr(VI)), necessitates a methodological approach grounded in the Margin of Exposure (MoE). This approach is particularly pertinent due to the BMDL_10_ of 1.0 mg Cr(VI)/kg bw/day serving as the point of departure (PD) for the combined incidence of adenomas and carcinomas in the murine small intestine^14^. Consequently, the objective of our original investigations resides in proposing a dual regulatory strategy for evaluating the human health risk associated with total chromium impurities in Marshmallow Root-based cough syrups available in Polish pharmacies. This approach encompasses:

1. Adherence to the principles outlined in the ICH Q3D (R1) guideline for elemental impurities.
2. The application of a Margin of Exposure (MoE) paradigm, tailored to both pediatric and adult populations, with specific emphasis on the mode of action of Cr(VI) (utilizing BMDL_10_ for Cr(VI) as the Point of Departure (PoD)).

**Figure 3:**
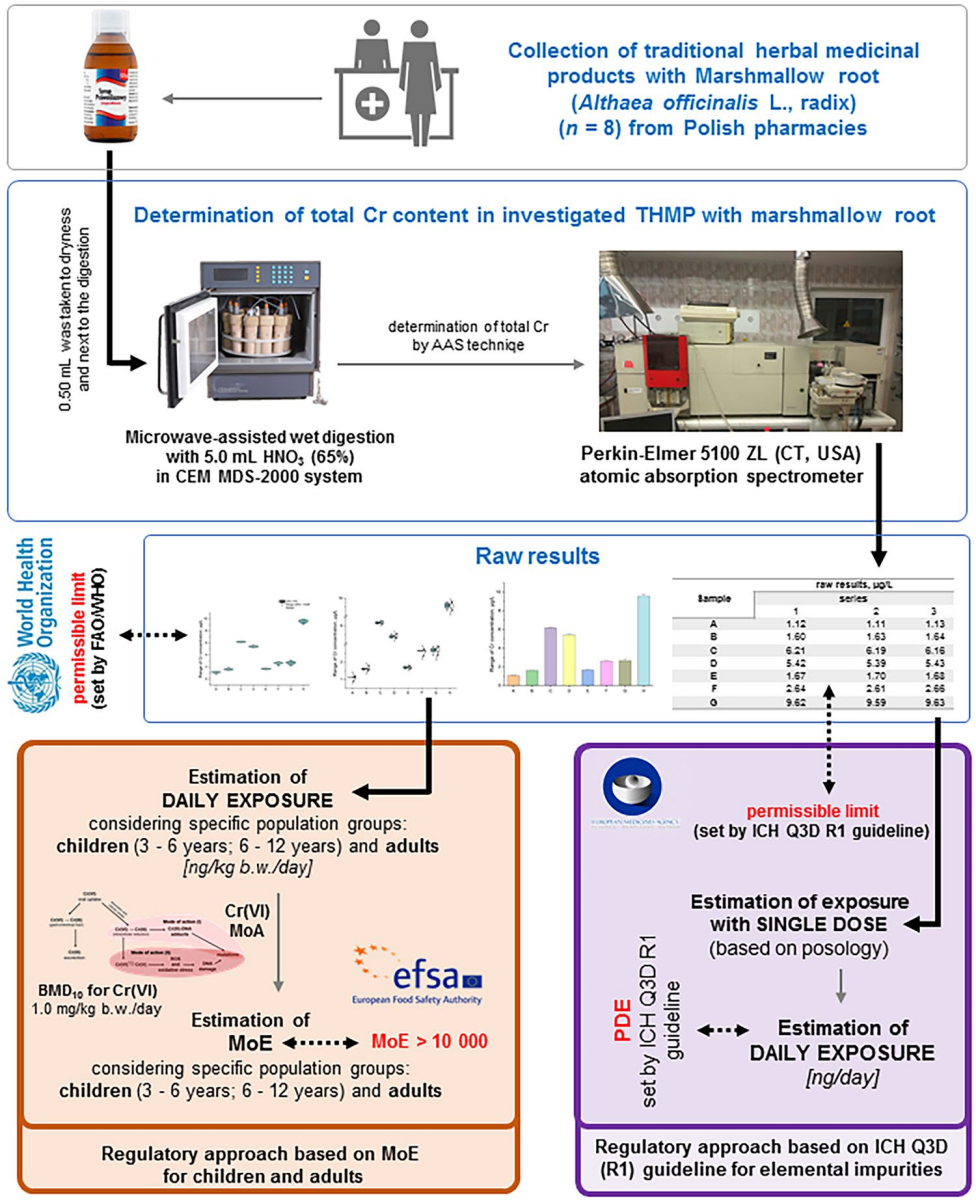
The basic workflow of the innovative double strategy in the Human Health Risk Assessment (HHRA) of Cr in THMP with Marshmallow Root (*Althaea officinalis*) available in Polish pharmacies.

The fundamental workflow of this bifurcated regulatory strategy, as applied to the HHRA of chromium within THMP featuring Marshmallow Root (*Althaea officinalis*), accessible through Polish pharmacies, is graphically depicted in Fig. 3.

## Results and discussion

### The first regulatory approach based on the ICH Q3D (R1) guideline for elemental impurities

The first regulatory approach of our proposed HHRA strategy is based on the ICH Q3D (R1) guideline for elemental impurities, which is described more clearly in separate steps (1–3).

#### Step 1: The measurement of total Cr impurities (raw results, determination of total Cr content in samples)

**Figure 4:**
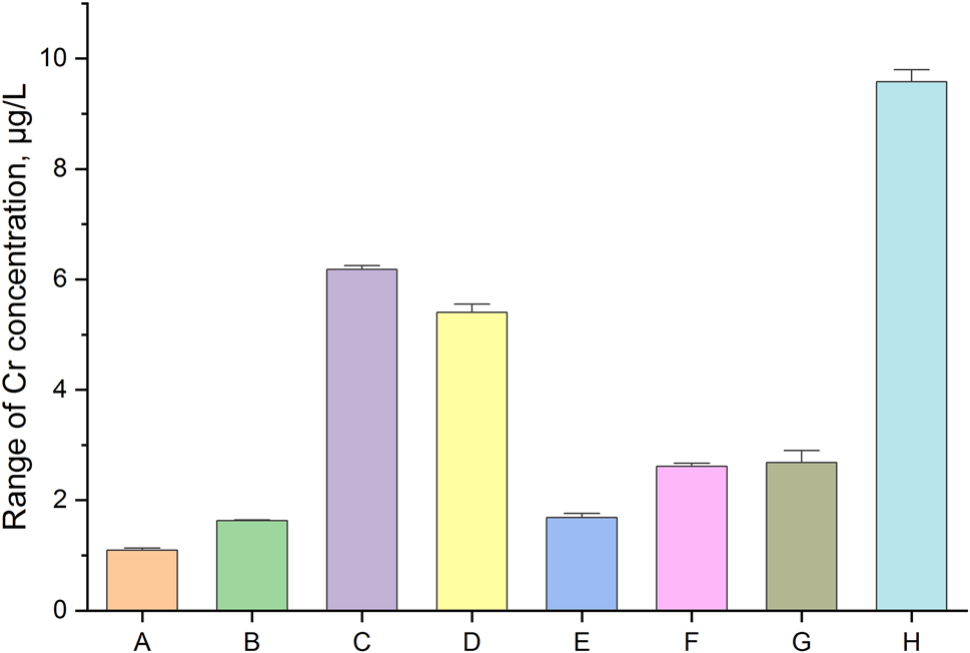
The profile for Cr impurities in the analyzed samples (A–H) as a column plot.

**Figure 5:**
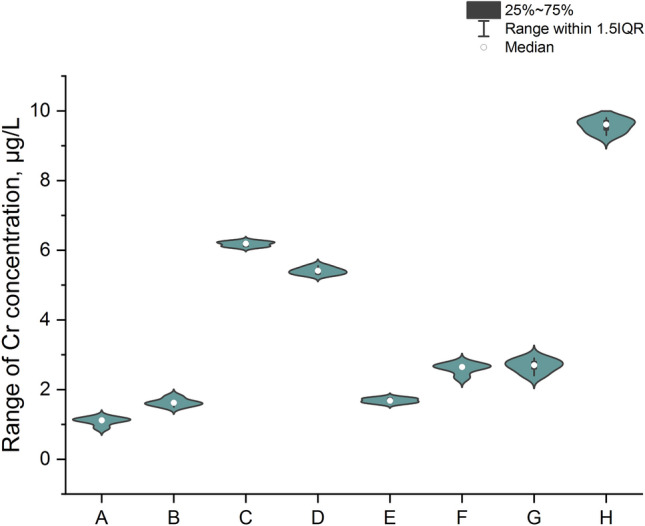
Profile of Cr impurities in analyzed samples (A–H) as a violin plot with box.

**Figure 6:**
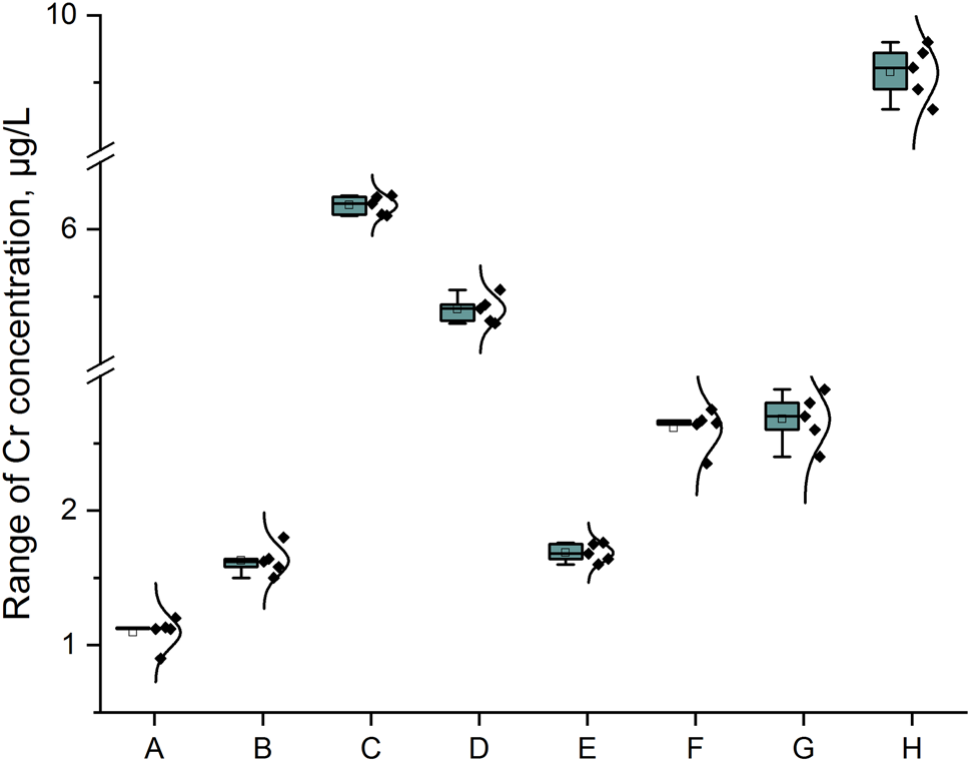
The profile for Cr impurities in the analyzed samples (A–H) as a box normal plot.

The first step of our study was the measurement of total Cr impurities in all analyzed samples. In this situation we decided to present results in raw format (Table 1; five independent replicates for each of three series for each sample) and graphical form – impurity profile: Fig. 4, simple column plot, Fig. 5, violin plot with box and Fig. 6, box normal.

**Table 1:** Raw results for the measurement of total Cr impurities in analyzed samples.

Sample	Raw results, µg/L		
	Series		
	1	2	3
A	1.12	1.11	1.13
B	1.60	1.63	1.64
C	6.21	6.19	6.16
D	5.42	5.39	5.43
E	1.67	1.70	1.68
F	2.64	2.61	2.66
G	9.62	9.59	9.63

The data form Table 1 and the Cr impurities profile (Fig. 4) in all the samples analyzed of the Marshmallow Root cough syrups (A–H) show that Cr was present in all the products analyzed products (minimum value to the maximum value was 1: 8.5). The results obtained are not coherent (range: 1.12–9.61 µg/L; median: 3.87 µg/L). The lowest levels were observed for the samples: A, B, and E. However, the highest levels were observed for the samples: H, C, and D. A similar level was observed for sample B (1.62 µg/L) and E (1.68 µg/L) and for sample F (2.64 µg/L) and G (2.70 µg/L). However, all products considered meet the requirements of the ICH Q3D guideline (1100 µg/g^1^). The violin plot with box (Fig. 5) and box normal plot confirms the symmetric distribution of the data and the illustrated range of the total Cr concentration in investigated products.

It is difficult to compare our results because we analyzed final pharmaceutical products. Only one article described by Divrikli et al.^17^ pointed the level of total Cr content in Marshmallow flower available in Turkey as 4.1 ± 0.1 µg/g. Therefore, our results are extremely lower than the pointed result of^18^.

#### Step 2: Estimation of Cr content with single oral dose

The second required step is to estimate the total chromium content in a single oral dose. For this purpose, the worst-case scenario should be applied based on doses of each analyzed product described by each manufacturer. In this approach, we assume the highest amount (mL) of orally administered drug in a single dose (based on Table S1) and additionally the results from the first step (Table 1). The estimation of Cr content in a single oral dose to which the patient is exposed for a single dose of the analyzed samples of cough syrups with Marshmallow Root is shown in Table 2.

**Table 2:** The estimation of Cr content in single oral dose of the analyzed samples of cough syrups with Marshmallow root.

Cough syrups with Marshmallow Root	Maximum single dose, mL	The estimation of Cr content in a single oral dose	
		ng/single dose	SD
A	15	16.8	0.62
B	15	24.35	1.24
C	15	92.8	0.98
D	15	81.2	0.74
E	15	25.25	0.64
F	15	39.55	0.71
G	15	40.45	0.65
H	5	48.07	0.78

SD-standard deviation.

It should be noted that the content of total Cr in a single dose, due to differences in the maximum single dose for each product, may significantly affect the final result.

#### Step 3: Estimation of Cr content in daily dose

The third step in our toxicological risk assessment of Cr is the estimation of Cr content in daily dose based on the results of the second step (estimation of Cr content in a single oral dose) and, in comparison, with the oral PDE value recommended by the ICH Q3D guideline (10,700 µg/day). The estimated values of Cr content in daily oral dose are presented in Table 3.

**Table 3:** Estimation of Cr content in daily oral dose of Marshmallow Root cough syrups (A–H).

cough syrups with Marshmallow Root	Frequency of use, times/day	The estimation of Cr content in daily oral dose	
		ng/day	SD
A	3	50.40	0.62
B	3	73.05	1.24
C	3	278.40	0.98
D	4	243.60	0.74
E	4	101.00	0.64
F	4	158.20	0.71
G	3	121.35	0.65
H	5	240.33	0.78

SD-standard deviation.

The analysis of the results obtained for the Cr content in daily oral dose shows that the results are incoherent (50.04–278.40 ng/day). Furthermore, the results obtained in the final steps are relatively low (< 300 ng/day). The comparison of the estimated results with the oral PDE value for Cr in the final drugs suggested by the ICH Q3D guideline (10,700 µg/day) show that all the products analysed are below this value (the highest result was 278.40 ng/day).

It should be underlined that we cannot compare the results obtained with any other because to our knowledge we first described estimation of Cr content in daily oral dose of cough syrups with Marshmallow Root.

#### The second regulatory approach based on Margin of Exposure (MoE) for children and adults

The second regulatory approach of our proposed HHRA strategy is the estimation of the margin of exposure (MoE) for Cr in all investigated samples, which is described more clearly in separate steps (1–2). This approach is a very universal and useful ‘toxicological tool’ that can be applied to impurities that are genotoxic and carcinogenic, regardless of their origin^19^. Hence, it can be applied for Cr assessment considering the genotoxicity of Cr(VI) as the worst-case scenario (WCS) (see Fig. 2).

#### Step 1: The estimation of Cr content in daily dose considering specific population groups: children (3–6 years; 6–12 years) and adults

First, the daily exposure values to a product (ng/kg bw/day) were estimated based on the amount applied and the frequency of application and the average weight of the specific population groups: children (3–6 years old; 6–12 years old) and adults. For this purpose, the estimation of Cr in the daily dose was carried out, depending on age and body weight for each population group (based on WHO growth standards^20^) was carry out. The obtained results of a daily dose of Cr depending on specific population groups in analysed samples (ng/kg b.w./day) are given in Table 4.

**Table 4:** The estimated of Cr in daily dose for each cough syrups with Marshmallow Root (A–H), depending on age and body weight for each specific population group (ng/kg b.w./day).

Specific population groups, age	Approximate body weight (kg)	THMP with Marshmallow Root							
		A	B	C	D	E	F	G	H
Children, 3–6 years old	15–23	3.36–2.191	4.870–3.176	18.560–12.104	16.240–10.591	6.733–4.391	10.547– 6.878	8.090–5.276	16.022–10.449
Children, 6–12 years of age	23–46	2.191–1.096	3.176–1.588	12.104–6.052	10.591–5.296	4.391– 2.196	6.878– 3.439	5.276–2.638	10.449–5.225
Adults	60	0.84	1.218	4.640	4.060	1.683	2.637	2.023	4.006

#### Step 2: The Margin of exposure (MoE) calculated for Cr in daily dose for each cough syrups with Marshmallow Root (A–H), depending on age and body weight for each specific population group

As mentioned above, MoE is a very universal and useful 'toxicological tool' that can be applied to impurities that are both genotoxic and carcinogenic, regardless of their origin^19^. MoE can be defined as the relationship between a point of departure (POD_sys_; usually historical NOAEL or BMDL_10_ values from oral studies) and an estimate of exposure—Eq. (1).

1 $${\text{MoE }} = {\text{ PODsys}}/{\text{Exposure}}$$

where POD_sys_—point of departure (mg/kg bw/day); Exposure—exposure (mg/kg bw/day).

In general, a MoE of 10,000 or more, if it is based on the BMDL_10_ of an animal carcinogenicity study, and taking into account general uncertainties in the interpretation, would be of low concern from a public health point of view and might reasonably be considered as a low priority for risk management actions. It has been assumed that the MoE value of 10,000 (or higher) is considered of low concern from a public health point of view with respect to the carcinogenic effect^19^. In this context, the most suitable PoD should be BMDL_10_ for Cr(VI). The justification is fact that Cr(VI) is most harmful for health—both MoA can occur and contribute to the genotoxic effects of Cr(VI) (see Fig. 2). Furthermore, since the sequence of adenoma-carcinoma is a well-recognised carcinogenesis pathway in the gastrointestinal tract, with a conservative approach, BMDL_10_ of 1.0 mg Cr (VI)/kg bw per day should be selected for combined adenomas or carcinomas of the small intestine in male and female mice as PoD for the estimation of MoE for neoplastic changes^19^. The calculated values of MoE for Cr in daily dose for each cough syrups with Marshmallow Root (A–H), depending on age and body weight for each specific population group are summarised in Table 5.

**Table 5:** Margin of exposure (MoE) calculated for Cr in daily dose for each cough syrup with Marshmallow Root (A–H), depending on age and body weight for each specific population group.

Specific population groups, age	Approximate body weight (kg)	THMP with Marshmallow Root							
		A	B	C	D	E	F	G	H
Children, 3–6 years old	15 – 23	297,619.05– 456,349.21	205,338.809 –314,852.841	53,879.310–82,614.943	61,576.35–94,417.077	148,514.85–227,722.77	94,816.68–145,385.58	123,609.39–189,534.40	62,413.31–95,700.41
Children, 6–12 years old	23 – 46	456,349.21–912,698.41	314,852.841–629,705.68	82,614.94–165,229.91	94,417.077–188,834.15	227,722.72–455,445.54	145,385.58–290,771.17	189,534.40–379,068.80	95,700.41–191,400.83
Adults	60	1,190,476.19	821,355.23	215,517.24	246,305.42	594,059.40	379,266.75	494,437.58	249,653.26

Despite conservative assumptions, the MoE values obtained for Cr in daily dose for each cough syrup with Marshmallow Root (A–H) are greater than 10,000, so exposure to Cr would not cause a health risk based on the MoE-based strategy.

## Conclusions

Chromium exhibits a range of oxidation states, with the trivalent (Cr(III)) and hexavalent (Cr(VI)) forms being the primary impurities found in pharmaceutical samples. Evidently, total chromium impurities were detected across all examined samples of Marshmallow Root cough syrups (A–H) available in Polish pharmacies. The observed values display a lack of consistency (spanning from 1.12 to 9.61 µg/L; median: 3.87 µg/L), suggesting variations in composition potentially stemming from distinct raw material sources employed by different manufacturers. However, the initial phase of the regulatory approach, aligned with the ICH Q3D (R1) guideline for elemental impurities, affirms that all assessed products adhere to the stipulated requirements set forth by the guideline (1100 µg/g^1^).

The subsequent phase permits the estimation of Cr content within a singular oral dose (ranging from 16.8 to 92.8 ng per single dose). While the aggregate Cr levels within a single dose appear relatively elevated in comparison to the raw data, they do not pose a threat to patients. The final stage conclusively verifies the safety of the examined cough syrups containing Marshmallow Root. The comparison of the estimated outcomes with the permissible daily exposure (PDE) value for chromium in final medicinal products, as recommended by the ICH Q3D (R1) guideline (10,700 µg/day), underscores that all analyzed products remain below this threshold (with the highest result recorded at 278.40 ng/day). The second regulatory approach, hinging on the MoE concept applicable to both pediatric and adult populations, reaffirms the safety of the scrutinized samples across all scenarios (all instances displaying MoE values significantly surpassing 10,000).

It is worth highlighting that this approach serves as a remarkably versatile and valuable ‘toxicological tool’, apt for application to impurities characterized by both genotoxic and carcinogenic properties, regardless of their origins. Given the potential occurrence of two distinct modes of action (MoAs) for orally administered Cr, both contributing to the genotoxic effects of Cr(VI), the utilization of this approach is crucial for aligning with contemporary regulatory requisites. Moreover, it is pertinent to emphasize that, while considering the speciation of Cr, further investigations utilizing the HPLC-ICP-MS technique could provide valuable insights. However, despite the unavailability of this costly and resource-intensive technique, we affirm the safety of the analyzed total Cr impurities, employing a MoE-based strategy for regulatory compliance, while accounting for worst-case scenarios of Cr(VI) impurities.

## Materials and methods

### Samples

The analyzed samples were cough syrups containing Marshmallow Root (*Althaea officinalis*), which are readily accessible within Polish pharmacies (*n* = 8). All eligible syrups featuring *Althaea officinalis* as officially registered traditional herbal medicinal products in Poland were encompassed within the research. It merits acknowledgment that each product was subjected to triple scrutiny. The majority of the examined products fell under the category of over-the-counter (OTC) medications. The procured items were sourced from local pharmacies situated in the Małopolska Voivodeship (Kraków, Niepołomice) and Podkarpackie voivodeship (Rzeszów) during the autumn period of 2022 (September–December). To ensure optimal conditions, the purchased products were assigned random codes (A–H) and were stored in a light-shielded room at a temperature ranging from 18 to 24 °C until the analytical procedures commenced. For comprehensive insight into the analyzed traditional herbal medicinal products featuring *Althaea officinalis* L., radix extracts (A–H), refer to Table S1.

### Chemicals and reagents

The experimental protocol relied upon demineralized water and concentrated nitric acid. Concentrated nitric acid (65%) of Merck SupraPur, Darmstadt, Germany, spectroscopic grade, was used for the preparation of solutions. Working solutions of chromium were prepared by diluting 1 mg/mL Cr(NO_3_)_3_ stock solutions (CertiPUR®) with demineralized water containing 0.5 mol/L nitric acid. The range of applied Cr working solutions spanned from 0.0 to 100.0 μg/L. A certified reference material (BCR-482; IRMM, Belgium) derived from lichen was obtained and utilized. Purge gas in the form of Argon (Ar) of 5N purity was employed.

### Sample preparation

The microwave oven MDS 2000 (CEM USA) along with a microwave-assisted digestion methodology was instrumental in the acid digestion of samples. Prior to Cr determination, each sample of traditional herbal medicinal products containing *Althaea officinalis* L., radix, underwent homogenization. A measure of 0.5 mL was extracted from each sample, transferred into Teflon vessels, and subjected to digestion using 5.0 mL of concentrated nitric acid (HNO_3_, 63%). The sealed vessels were subsequently subjected to microwave irradiation over a span of 2 h. Post-microwave treatment, the samples were allowed to cool to room temperature (25 °C), after which their final volume was adjusted to 20 mL. These cooled samples were then stored in plastic containers as stock sample solutions until further analysis. A quintuple replication approach was maintained for all samples, augmenting the precision of the outcomes.

### Determination of total chromium content in samples

For the determination of total Cr content, the Perkin-Elmer 5100 ZL atomic absorption spectrometer (Perkin-Elmer, Norwalk, CT, USA), equipped with Zeeman background correction and operating under the electrothermal atomization (ET AAS technique), was employed. The specific time–temperature programme employed in the graphite furnace atomic absorption spectrometer is elucidated in Table 6. The pertinent instrumental parameters are consolidated in Table 7.

**Table 6:** Time–temperature program in the graphite furnace atomic absorption spectrometer for total Cr determination.

Step	Temperature, °C	Ramp, s	Hold, s
1	110	5	15
2	180	30	10
3	450	1	5
4	500	5	5
5	1500	15	30
6	2450	0	5
7	2500	1	2

**Table 7:** The applied instrumental conditions for Cr determination.

Parameter	Value
Emission source	Cr hollow cathode lamp
Wavelength, nm	357.9
Lamp current, mA	0.5
Slit width, nm	0.7
Sample volume, µL	40
Integrated absorbance (peak area)	Applied
Background correction	Zeeman background correction
Atomization	Electrothermal atomization (ETAAS technique)

### The analytical calibration procedure and quality approaches

The calibration function was constructed based on a series of working solutions of Cr (5.0, 10, 50.0, 100.0, and 150.0 μg/L). These solutions were meticulously prepared from stock solutions of 1000 μg/mL (Cr(NO_3_)_3_; CertiPUR®), employing ultrapure demineralized water within a 0.5 mol/L nitric acid matrix. The obtained correlation coefficient yielded a satisfactory value (R = 0.999). Recovery assessments were performed, resulting in a recovery rate of 98.53% for BCR-482; IRMM, Belgium, and 98.2% for Corn Flour, INCT-CF-3. This recovery rate was calculated as the ratio of the determined level to the known quantity of Cr, expressed as a percentage. The certified Cr value stood at 0.134 mg/kg, while the measured value amounted to 0.136 mg/kg.

The limit of detection (LOD), as defined by (3 SD)/a, where SD represents the standard deviation corresponding to ten blank injections and ‘a’ symbolizes the slope of the calibration function, was determined to be 1.67 µg/L for BCR-482; IRMM, Belgium, as well as for Corn Flour, INCT-CF-3. Correspondingly, the limit of quantification (LOQ), defined by (10 SD)/a, yielded a value of 4.95 µg/L for both BCR-482; IRMM, Belgium, and Corn Flour, INCT-CF-3. As mentioned in the “Chemicals and reagents” section, the certified reference material was prepared from lichen (BCR-482; IRMM, Belgium) and Corn Flour, INCT-CF-3. Prior to analysis, the samples were subjected to a drying process at 70 °C for a duration of 24 h. Post-drying, the samples were transformed into a solution via microwave digestion, facilitated by a programmable microwave oven (MDS-2000; CEM Corp., Mattews, USA). The procedure involved introducing 5 mL of nitric acid (65%) to 300 mg of the certified reference material within Teflon reaction vessels, allowing a 24-h pre-digestion phase. Subsequent to pre-digestion, the samples underwent full digestion. Following cooling of the reaction vessels, the contents were quantitatively transferred to Sarstedt vessels and supplemented with demineralized water to achieve a final volume of 15 mL. Samples prepared according to this protocol were subjected to analysis using the Perkin-Elmer 5100 ZL atomic absorption spectrometer, employing the graphite furnace mode.

The anticipated Cr value for BCR-482; IRMM, Belgium was 40.9 ± 1.4 mg/kg, while the obtained value stood at 38.05 ± 0.23 mg/kg. For Corn Flour, INCT-CF-3, the projected Cr value was 0.052 ± 0.009 mg/kg, and the measured value coincided at 0.050 ± 0.009 mg/kg. The analysis of the aforementioned certified reference materials served as a crucial means of evaluating the traceability and accuracy of the results. The applied methodology closely mirrored our preceding studies, employing the same analytical apparatus^15–17^.

### The innovative regulatory strategy in the human health risk assessment of total chromium impurities in cough syrups with Marshmallow Root (*Althaea officinalis*) available in Polish pharmacies

In our study we have two important regulatory issues: requirements of ICH Q3D (R1) guideline for elemental impurities and exposure for specific population groups. Hence, the double HHRA is needed. It should be emphasized that the idea of this work is not determination of the total Cr content in the investigated THMP samples (this is not analytical work). This is only the basis that is used for the comprehensive HHRA including two approaches for regulatory purposes:

1. the requirements of ICH Q3D (R1) guideline for elemental impurities;
2. Margin of exposure (MoE) for children and adults with special emphasis on Cr(VI) mode of action (BMDL10 for Cr(VI) as PoD).

The basic workflow of the applied double regulatory strategy in the HHRA of Cr in THMP with Marshmallow Root (*Althaea officinalis*) available in Polish pharmacies is shown schematically on Fig. 3.

### Data analysis

The results of five independent replicates were expressed as the mean ± standard deviation. Obtained results were analysed using statistical softwares: Excel 2010 (Microsoft Office) and Origin 2021 Pro the Ultimate Software for Graphing and Analysis (OriginLab Corporation, One Roundhouse Plaza, Suite 303, Northampton, MA 01060, USA) licensed by the Jagiellonian University in Krakow.

### Supplementary Information

Supplementary Table S1.

## Data Availability

The datasets used and/or analyzed during the current study are available from the corresponding authors on reasonable request.
