# Supplementary material for: The comprehensive strategy in the human health risk assessment of total chromium impurities in cough syrups with Marshmallow Root (Althaea officinalis) available in Polish pharmacies: regulatory aspects and special emphasis on Cr(VI) mode of action
Source: Sci Rep. 2024 Mar 4;14:5293. doi: 10.1038/s41598-024-56057-7 (PMC10912266; doi:10.1038/s41598-024-56057-7)
Supplement: Supplementary file 1 — Supplementary Table S1. [file 41598_2024_56057_MOESM1_ESM.rtf]

Table S1. The detailed information about investigated traditional herbal medicinal products with Althaea officinalis L., radix (marshmallow root) purchased in pharmacies from Poland.
Descriptor	Sample	
	A	B	C	D	E	F	G	H	
Herbal plant	A. officinalis L.	A. officinalis L.	A. officinalis L.	A. officinalis L.	A. officinalis L.	A. officinalis L.	A. officinalis L.	A. officinalis L.	
Part of herbal plant	root	root	root	root	root	root	root	root	
Kind of traditional herbal medicinal product 	syrup	syrup	syrup	syrup	syrup	syrup	syrup	syrup	
Preparation	Liquid extract 
(DER 1:1), extraction solvent ethanol 25% (V/V)	Liquid extract 
(DER 1:1), extraction solvent ethanol 30% (V/V)	Liquid extract 
(DER 1:1), extraction solvent ethanol 20% (V/V)	Liquid extract 
(DER 1:1), extraction solvent ethanol 25% (V/V)	Liquid extract 
(DER 1:1), extraction solvent ethanol 25% (V/V)	Liquid extract 
(DER 1:1), extraction solvent ethanol 25% (V/V)	Liquid extract 
(DER 1:1), extraction solvent ethanol 25% (V/V)	Liquid extract 
(DER 1:1), extraction solvent ethanol 25% (V/V)	
Density, g/mL	1.29	1.31	1.26	1.28	1.32	1.30	1.295	1.33	
Lot number	20920	02AF0620	01AF0820	01AF0920	21220	61020	10420	454201	
License	IL-0692/LN	12180	no applicable	no applicable	IL-5954/LN	IL-4746/LN	8147	no applicable	
classification	OTC	OTC	diet supplement	diet supplement	OTC	OTC	OTC	medical product	
Amount 
of API
in 10 mL 
of syrup	0.33	0.27	0.29	0.21	0.28	0.31	0.30	0.25	
Single dose	Oral use: 
15 mL, 
3 times daily	Oral use: 
10-15 mL, 
3 times daily	Oral use: 
15 mL, 
3 times daily	Oral use: 
15 mL, 
3 times daily	Oral use: 
15 mL, 
3-4 times daily	Oral use: 
15 mL, 
3-4 times daily	Oral use: 
15 mL, 
3 times daily	Oral use: 
5 mL, 
5 times daily	
Place of purchase	Kraków	Niepo³omice	Rzeszów	Rzeszów	Kraków	Niepo³omice	Rzeszów	Kraków	
